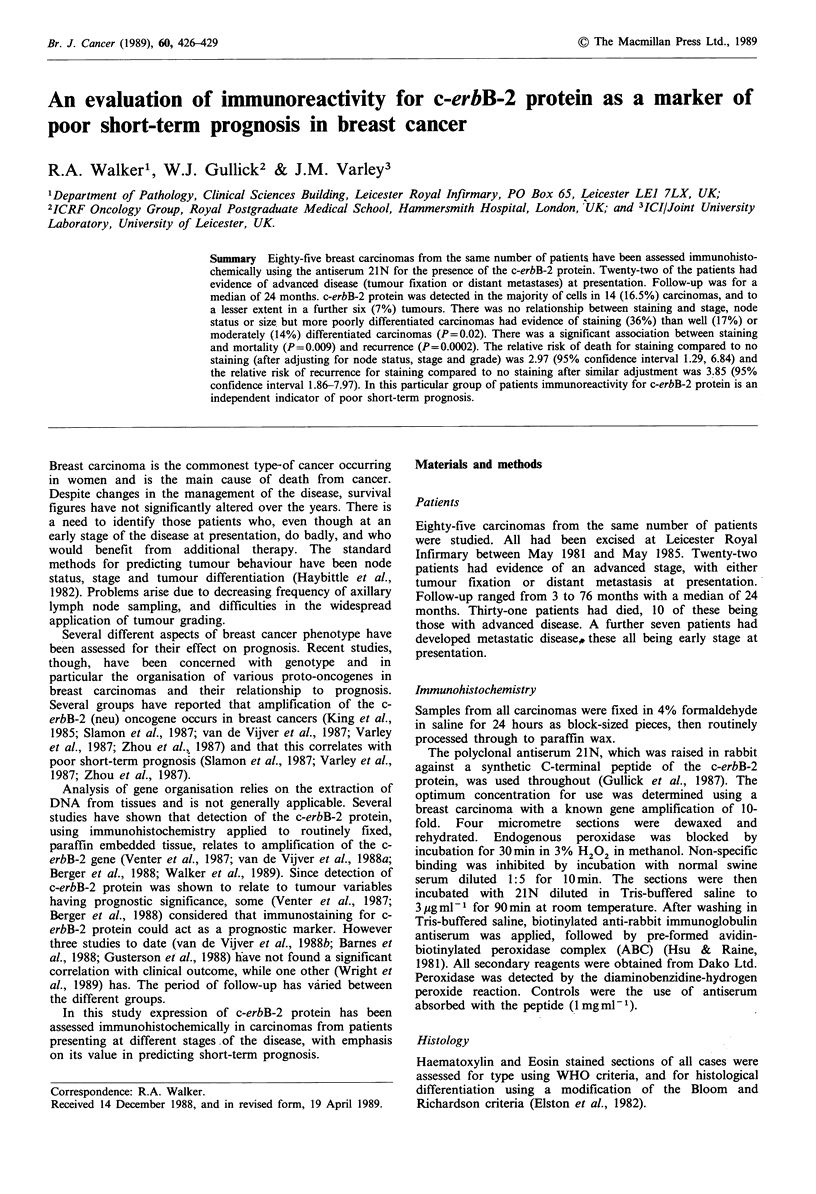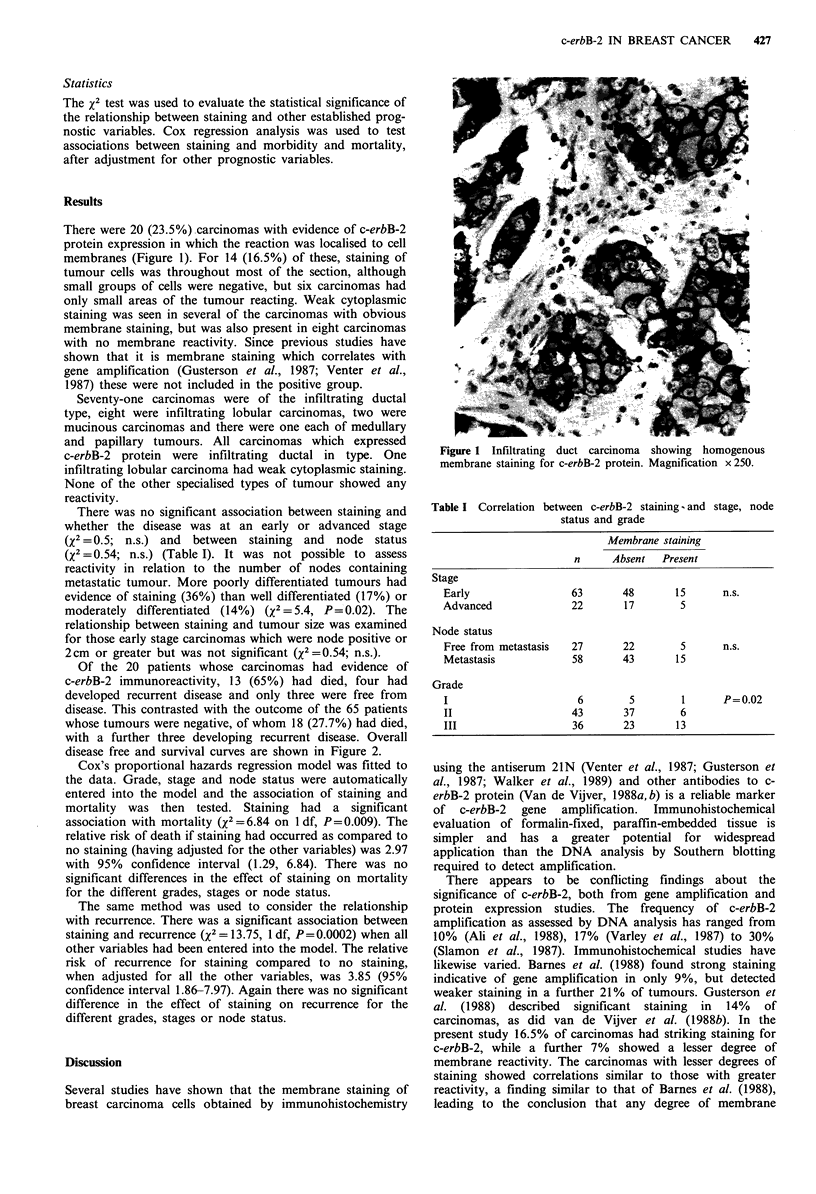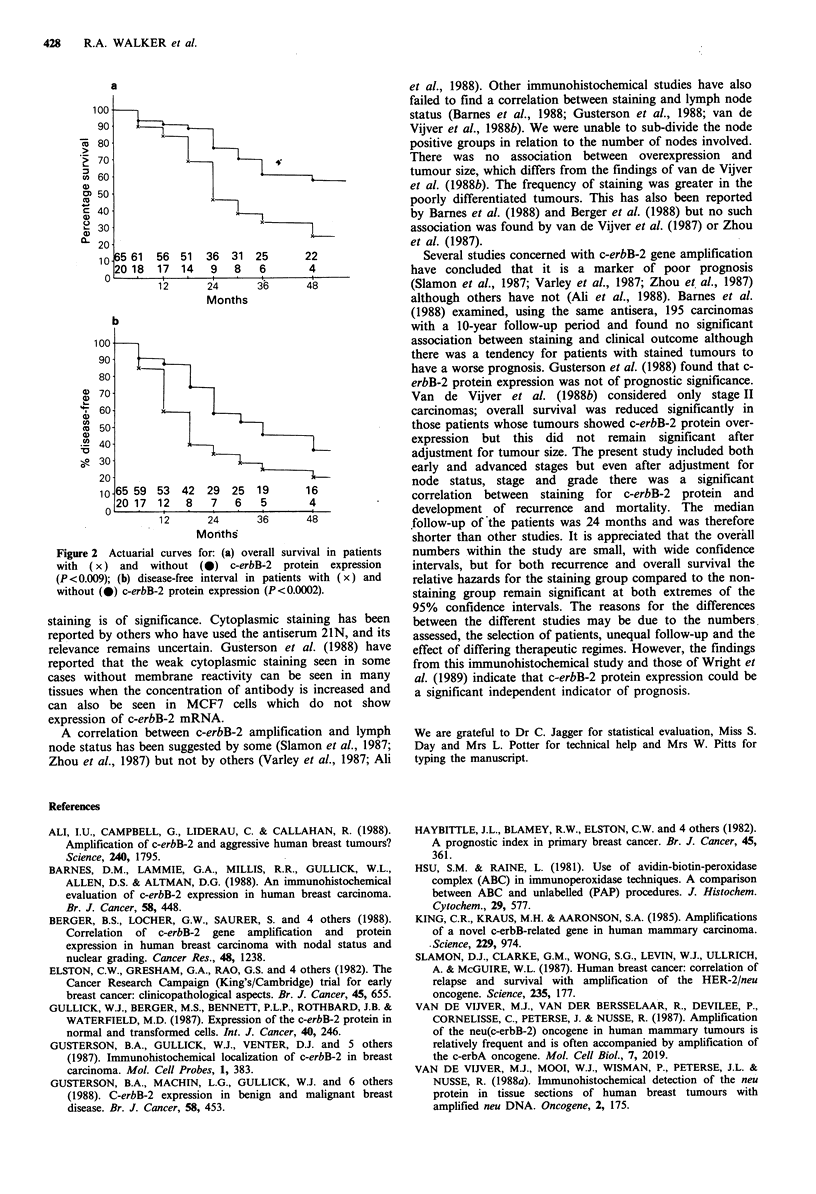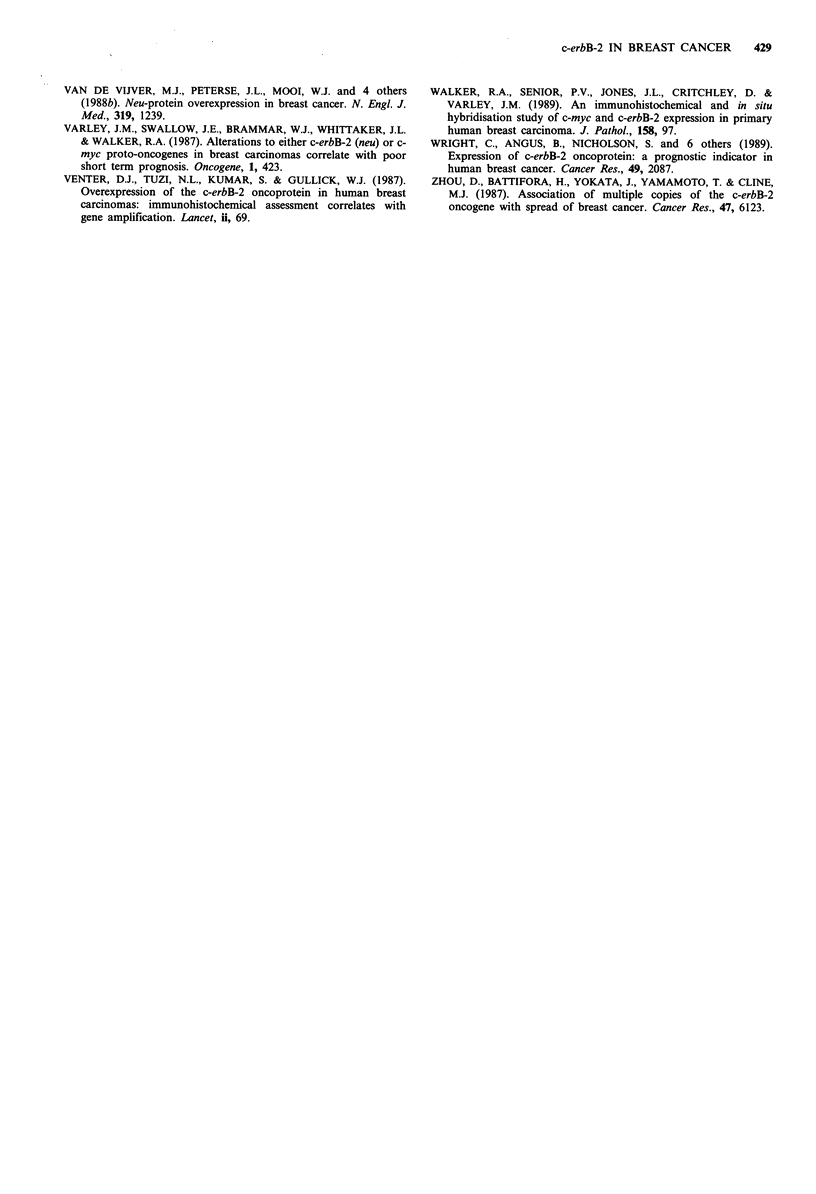# An evaluation of immunoreactivity for c-erbB-2 protein as a marker of poor short-term prognosis in breast cancer.

**DOI:** 10.1038/bjc.1989.299

**Published:** 1989-09

**Authors:** R. A. Walker, W. J. Gullick, J. M. Varley

**Affiliations:** Department of Pathology, Leicester Royal Infirmary, UK.

## Abstract

**Images:**


Br. J. Cancer (1989), **60**, 426–429 © The Macmillan Press Ltd., 1989

### An evaluation of immunoreactivity for c-*erb*B-2 protein as a marker of poor short-term prognosis in breast cancer

R.A. Walker[1], W.J. Gullick[2] & J.M. Varley[3]

[1]*Department of Pathology, Clinical Sciences Building, Leicester Royal Infirmary, PO Box 65, Leicester LE1 7LX, UK;* [2]*ICRF Oncology Group, Royal Postgraduate Medical School, Hammersmith Hospital, London, UK; and* [3]*ICI/Joint University Laboratory, University of Leicester, UK.*

**Summary** Eighty-five breast carcinomas from the same number of patients have been assessed immunohistochemically using the antiserum 21N for the presence of the c-*erb*B-2 protein. Twenty-two of the patients had evidence of advanced disease (tumour fixation or distant metastases) at presentation. Follow-up was for a median of 24 months. c-*erb*B-2 protein was detected in the majority of cells in 14 (16.5%) carcinomas, and to a lesser extent in a further six (7%) tumours. There was no relationship between staining and stage, node status or size but more poorly differentiated carcinomas had evidence of staining (36%) than well (17%) or moderately (14%) differentiated carcinomas ($P=0.02$). There was a significant association between staining and mortality ($P=0.009$) and recurrence ($P=0.0002$). The relative risk of death for staining compared to no staining (after adjusting for node status, stage and grade) was 2.97 (95% confidence interval 1.29, 6.84) and the relative risk of recurrence for staining compared to no staining after similar adjustment was 3.85 (95% confidence interval 1.86–7.97). In this particular group of patients immunoreactivity for c-*erb*B-2 protein is an independent indicator of poor short-term prognosis.

Breast carcinoma is the commonest type of cancer occurring in women and is the main cause of death from cancer. Despite changes in the management of the disease, survival figures have not significantly altered over the years. There is a need to identify those patients who, even though at an early stage of the disease at presentation, do badly, and who would benefit from additional therapy. The standard methods for predicting tumour behaviour have been node status, stage and tumour differentiation (Haybittle *et al.*, 1982). Problems arise due to decreasing frequency of axillary lymph node sampling, and difficulties in the widespread application of tumour grading.

Several different aspects of breast cancer phenotype have been assessed for their effect on prognosis. Recent studies, though, have been concerned with genotype and in particular the organisation of various proto-oncogenes in breast carcinomas and their relationship to prognosis. Several groups have reported that amplification of the c-*erb*B-2 (neu) oncogene occurs in breast cancers (King *et al.*, 1985; Slamon *et al.*, 1987; van de Vijver *et al.*, 1987; Varley *et al.*, 1987; Zhou *et al.*, 1987) and that this correlates with poor short-term prognosis (Slamon *et al.*, 1987; Varley *et al.*, 1987; Zhou *et al.*, 1987).

Analysis of gene organisation relies on the extraction of DNA from tissues and is not generally applicable. Several studies have shown that detection of the c-*erb*B-2 protein, using immunohistochemistry applied to routinely fixed, paraffin embedded tissue, relates to amplification of the c-*erb*B-2 gene (Venter *et al.*, 1987; van de Vijver *et al.*, 1988*a*; Berger *et al.*, 1988; Walker *et al.*, 1989). Since detection of c-*erb*B-2 protein was shown to relate to tumour variables having prognostic significance, some (Venter *et al.*, 1987; Berger *et al.*, 1988) considered that immunostaining for c-*erb*B-2 protein could act as a prognostic marker. However three studies to date (van de Vijver *et al.*, 1988*b*; Barnes *et al.*, 1988; Gusterson *et al.*, 1988) have not found a significant correlation with clinical outcome, while one other (Wright *et al.*, 1989) has. The period of follow-up has varied between the different groups.

In this study expression of c-*erb*B-2 protein has been assessed immunohistochemically in carcinomas from patients presenting at different stages of the disease, with emphasis on its value in predicting short-term prognosis.

Correspondence: R.A. Walker.
Received 14 December 1988, and in revised form, 19 April 1989.

### Materials and methods

### *Patients*

Eighty-five carcinomas from the same number of patients were studied. All had been excised at Leicester Royal Infirmary between May 1981 and May 1985. Twenty-two patients had evidence of an advanced stage, with either tumour fixation or distant metastasis at presentation. Follow-up ranged from 3 to 76 months with a median of 24 months. Thirty-one patients had died, 10 of these being those with advanced disease. A further seven patients had developed metastatic disease, these all being early stage at presentation.

### *Immunohistochemistry*

Samples from all carcinomas were fixed in 4% formaldehyde in saline for 24 hours as block-sized pieces, then routinely processed through to paraffin wax.

The polyclonal antiserum 21N, which was raised in rabbit against a synthetic C-terminal peptide of the c-*erb*B-2 protein, was used throughout (Gullick *et al.*, 1987). The optimum concentration for use was determined using a breast carcinoma with a known gene amplification of 10-fold. Four micrometre sections were dewaxed and rehydrated. Endogenous peroxidase was blocked by incubation for 30 min in 3% $H_2O_2$ in methanol. Non-specific binding was inhibited by incubation with normal swine serum diluted 1:5 for 10 min. The sections were then incubated with 21N diluted in Tris-buffered saline to $3\,\mu g\,ml^{-1}$ for 90 min at room temperature. After washing in Tris-buffered saline, biotinylated anti-rabbit immunoglobulin antiserum was applied, followed by pre-formed avidin-biotinylated peroxidase complex (ABC) (Hsu & Raine, 1981). All secondary reagents were obtained from Dako Ltd. Peroxidase was detected by the diaminobenzidine-hydrogen peroxide reaction. Controls were the use of antiserum absorbed with the peptide ($1\,mg\,ml^{-1}$).

### *Histology*

Haematoxylin and Eosin stained sections of all cases were assessed for type using WHO criteria, and for histological differentiation using a modification of the Bloom and Richardson criteria (Elston *et al.*, 1982).

*Statistics*

The $\chi^2$ test was used to evaluate the statistical significance of the relationship between staining and other established prognostic variables. Cox regression analysis was used to test associations between staining and morbidity and mortality, after adjustment for other prognostic variables.

### Results

There were 20 (23.5%) carcinomas with evidence of c-*erb*B-2 protein expression in which the reaction was localised to cell membranes (Figure 1). For 14 (16.5%) of these, staining of tumour cells was throughout most of the section, although small groups of cells were negative, but six carcinomas had only small areas of the tumour reacting. Weak cytoplasmic staining was seen in several of the carcinomas with obvious membrane staining, but was also present in eight carcinomas with no membrane reactivity. Since previous studies have shown that it is membrane staining which correlates with gene amplification (Gusterson *et al.*, 1987; Venter *et al.*, 1987) these were not included in the positive group.

Seventy-one carcinomas were of the infiltrating ductal type, eight were infiltrating lobular carcinomas, two were mucinous carcinomas and there were one each of medullary and papillary tumours. All carcinomas which expressed c-*erb*B-2 protein were infiltrating ductal in type. One infiltrating lobular carcinoma had weak cytoplasmic staining. None of the other specialised types of tumour showed any reactivity.

There was no significant association between staining and whether the disease was at an early or advanced stage ($\chi^2 = 0.5$; n.s.) and between staining and node status ($\chi^2 = 0.54$; n.s.) (Table I). It was not possible to assess reactivity in relation to the number of nodes containing metastatic tumour. More poorly differentiated tumours had evidence of staining (36%) than well differentiated (17%) or moderately differentiated (14%) ($\chi^2 = 5.4$, $P = 0.02$). The relationship between staining and tumour size was examined for those early stage carcinomas which were node positive or 2 cm or greater but was not significant ($\chi^2 = 0.54$; n.s.).

Of the 20 patients whose carcinomas had evidence of c-*erb*B-2 immunoreactivity, 13 (65%) had died, four had developed recurrent disease and only three were free from disease. This contrasted with the outcome of the 65 patients whose tumours were negative, of whom 18 (27.7%) had died, with a further three developing recurrent disease. Overall disease free and survival curves are shown in Figure 2.

Cox's proportional hazards regression model was fitted to the data. Grade, stage and node status were automatically entered into the model and the association of staining and mortality was then tested. Staining had a significant association with mortality ($\chi^2 = 6.84$ on 1 df, $P = 0.009$). The relative risk of death if staining had occurred as compared to no staining (having adjusted for the other variables) was 2.97 with 95% confidence interval (1.29, 6.84). There was no significant differences in the effect of staining on mortality for the different grades, stages or node status.

The same method was used to consider the relationship with recurrence. There was a significant association between staining and recurrence ($\chi^2 = 13.75$, 1 df, $P = 0.0002$) when all other variables had been entered into the model. The relative risk of recurrence for staining compared to no staining, when adjusted for all the other variables, was 3.85 (95% confidence interval 1.86–7.97). Again there was no significant difference in the effect of staining on recurrence for the different grades, stages or node status.

### Discussion

Several studies have shown that the membrane staining of breast carcinoma cells obtained by immunohistochemistry using the antiserum 21N (Venter *et al.*, 1987; Gusterson *et al.*, 1987; Walker *et al.*, 1989) and other antibodies to c-*erb*B-2 protein (Van de Vijver, 1988*a,b*) is a reliable marker of c-*erb*B-2 gene amplification. Immunohistochemical evaluation of formalin-fixed, paraffin-embedded tissue is simpler and has a greater potential for widespread application than the DNA analysis by Southern blotting required to detect amplification.

There appears to be conflicting findings about the significance of c-*erb*B-2, both from gene amplification and protein expression studies. The frequency of c-*erb*B-2 amplification as assessed by DNA analysis has ranged from 10% (Ali *et al.*, 1988), 17% (Varley *et al.*, 1987) to 30% (Slamon *et al.*, 1987). Immunohistochemical studies have likewise varied. Barnes *et al.* (1988) found strong staining indicative of gene amplification in only 9%, but detected weaker staining in a further 21% of tumours. Gusterson *et al.* (1988) described significant staining in 14% of carcinomas, as did van de Vijver *et al.* (1988*b*). In the present study 16.5% of carcinomas had striking staining for c-*erb*B-2, while a further 7% showed a lesser degree of membrane reactivity. The carcinomas with lesser degrees of staining showed correlations similar to those with greater reactivity, a finding similar to that of Barnes *et al.* (1988), leading to the conclusion that any degree of membrane

**Figure 1** Infiltrating duct carcinoma showing homogenous membrane staining for c-*erb*B-2 protein. Magnification ×250.

**Table I** Correlation between c-*erb*B-2 staining and stage, node status and grade

| | *n* | Membrane staining | | |
|---|---|---|---|---|
| | | *Absent* | *Present* | |
| Stage | | | | |
| Early | 63 | 48 | 15 | n.s. |
| Advanced | 22 | 17 | 5 | |
| Node status | | | | |
| Free from metastasis | 27 | 22 | 5 | n.s. |
| Metastasis | 58 | 43 | 15 | |
| Grade | | | | |
| I | 6 | 5 | 1 | $P = 0.02$ |
| II | 43 | 37 | 6 | |
| III | 36 | 23 | 13 | |

**Figure 2** Actuarial curves for: (**a**) overall survival in patients with (×) and without (●) c-*erb*B-2 protein expression ($P<0.009$); (**b**) disease-free interval in patients with (×) and without (●) c-*erb*B-2 protein expression ($P<0.0002$).

staining is of significance. Cytoplasmic staining has been reported by others who have used the antiserum 21N, and its relevance remains uncertain. Gusterson *et al.* (1988) have reported that the weak cytoplasmic staining seen in some cases without membrane reactivity can be seen in many tissues when the concentration of antibody is increased and can also be seen in MCF7 cells which do not show expression of c-*erb*B-2 mRNA.

A correlation between c-*erb*B-2 amplification and lymph node status has been suggested by some (Slamon *et al.*, 1987; Zhou *et al.*, 1987) but not by others (Varley *et al.*, 1987; Ali *et al.*, 1988). Other immunohistochemical studies have also failed to find a correlation between staining and lymph node status (Barnes *et al.*, 1988; Gusterson *et al.*, 1988; van de Vijver *et al.*, 1988*b*). We were unable to sub-divide the node positive groups in relation to the number of nodes involved. There was no association between overexpression and tumour size, which differs from the findings of van de Vijver *et al.* (1988*b*). The frequency of staining was greater in the poorly differentiated tumours. This has also been reported by Barnes *et al.* (1988) and Berger *et al.* (1988) but no such association was found by van de Vijver *et al.* (1987) or Zhou *et al.* (1987).

Several studies concerned with c-*erb*B-2 gene amplification have concluded that it is a marker of poor prognosis (Slamon *et al.*, 1987; Varley *et al.*, 1987; Zhou *et al.*, 1987) although others have not (Ali *et al.*, 1988). Barnes *et al.* (1988) examined, using the same antisera, 195 carcinomas with a 10-year follow-up period and found no significant association between staining and clinical outcome although there was a tendency for patients with stained tumours to have a worse prognosis. Gusterson *et al.* (1988) found that c-*erb*B-2 protein expression was not of prognostic significance. Van de Vijver *et al.* (1988*b*) considered only stage II carcinomas; overall survival was reduced significantly in those patients whose tumours showed c-*erb*B-2 protein overexpression but this did not remain significant after adjustment for tumour size. The present study included both early and advanced stages but even after adjustment for node status, stage and grade there was a significant correlation between staining for c-*erb*B-2 protein and development of recurrence and mortality. The median follow-up of the patients was 24 months and was therefore shorter than other studies. It is appreciated that the overall numbers within the study are small, with wide confidence intervals, but for both recurrence and overall survival the relative hazards for the staining group compared to the non-staining group remain significant at both extremes of the 95% confidence intervals. The reasons for the differences between the different studies may be due to the numbers assessed, the selection of patients, unequal follow-up and the effect of differing therapeutic regimes. However, the findings from this immunohistochemical study and those of Wright *et al.* (1989) indicate that c-*erb*B-2 protein expression could be a significant independent indicator of prognosis.

We are grateful to Dr C. Jagger for statistical evaluation, Miss S. Day and Mrs L. Potter for technical help and Mrs W. Pitts for typing the manuscript.

### References

ALI, I.U., CAMPBELL, G., LIDERAU, C. & CALLAHAN, R. (1988). Amplification of c-*erb*B-2 and aggressive human breast tumours? *Science*, **240**, 1795.

BARNES, D.M., LAMMIE, G.A., MILLIS, R.R., GULLICK, W.L., ALLEN, D.S. & ALTMAN, D.G. (1988). An immunohistochemical evaluation of c-*erb*B-2 expression in human breast carcinoma. *Br. J. Cancer*, **58**, 448.

BERGER, B.S., LOCHER, G.W., SAURER, S. and 4 others (1988). Correlation of c-*erb*B-2 gene amplification and protein expression in human breast carcinoma with nodal status and nuclear grading. *Cancer Res.*, **48**, 1238.

ELSTON, C.W., GRESHAM, G.A., RAO, G.S. and 4 others (1982). The Cancer Research Campaign (King's/Cambridge) trial for early breast cancer: clinicopathological aspects. *Br. J. Cancer*, **45**, 655.

GULLICK, W.J., BERGER, M.S., BENNETT, P.L.P., ROTHBARD, J.B. & WATERFIELD, M.D. (1987). Expression of the c-*erb*B-2 protein in normal and transformed cells. *Int. J. Cancer*, **40**, 246.

GUSTERSON, B.A., GULLICK, W.J., VENTER, D.J. and 5 others (1987). Immunohistochemical localization of c-*erb*B-2 in breast carcinoma. *Mol. Cell Probes*, **1**, 383.

GUSTERSON, B.A., MACHIN, L.G., GULLICK, W.J. and 6 others (1988). C-*erb*B-2 expression in benign and malignant breast disease. *Br. J. Cancer*, **58**, 453.

HAYBITTLE, J.L., BLAMEY, R.W., ELSTON, C.W. and 4 others (1982). A prognostic index in primary breast cancer. *Br. J. Cancer*, **45**, 361.

HSU, S.M. & RAINE, L. (1981). Use of avidin-biotin-peroxidase complex (ABC) in immunoperoxidase techniques. A comparison between ABC and unlabelled (PAP) procedures. *J. Histochem. Cytochem.*, **29**, 577.

KING, C.R., KRAUS, M.H. & AARONSON, S.A. (1985). Amplifications of a novel c-erbB-related gene in human mammary carcinoma. *Science*, **229**, 974.

SLAMON, D.J., CLARKE, G.M., WONG, S.G., LEVIN, W.J., ULLRICH, A. & McGUIRE, W.L. (1987). Human breast cancer: correlation of relapse and survival with amplification of the HER-2/*neu* oncogene. *Science*, **235**, 177.

VAN DE VIJVER, M.J., VAN DER BERSSELAAR, R., DEVILEE, P., CORNELISSE, C., PETERSE, J. & NUSSE, R. (1987). Amplification of the neu(c-erbB-2) oncogene in human mammary tumours is relatively frequent and is often accompanied by amplification of the c-erbA oncogene. *Mol. Cell Biol.*, **7**, 2019.

VAN DE VIJVER, M.J., MOOI, W.J., WISMAN, P., PETERSE, J.L. & NUSSE, R. (1988*a*). Immunohistochemical detection of the *neu* protein in tissue sections of human breast tumours with amplified *neu* DNA. *Oncogene*, **2**, 175.

VAN DE VIJVER, M.J., PETERSE, J.L., MOOI, W.J. and 4 others (1988*b*). *Neu*-protein overexpression in breast cancer. *N. Engl. J. Med.*, **319**, 1239.

VARLEY, J.M., SWALLOW, J.E., BRAMMAR, W.J., WHITTAKER, J.L. & WALKER, R.A. (1987). Alterations to either c-*erb*B-2 (*neu*) or c-*myc* proto-oncogenes in breast carcinomas correlate with poor short term prognosis. *Oncogene*, **1**, 423.

VENTER, D.J., TUZI, N.L., KUMAR, S. & GULLICK, W.J. (1987). Overexpression of the c-*erb*B-2 oncoprotein in human breast carcinomas: immunohistochemical assessment correlates with gene amplification. *Lancet*, **ii**, 69.

WALKER, R.A., SENIOR, P.V., JONES, J.L., CRITCHLEY, D. & VARLEY, J.M. (1989). An immunohistochemical and *in situ* hybridisation study of c-*myc* and c-*erb*B-2 expression in primary human breast carcinoma. *J. Pathol.*, **158**, 97.

WRIGHT, C., ANGUS, B., NICHOLSON, S. and 6 others (1989). Expression of c-*erb*B-2 oncoprotein: a prognostic indicator in human breast cancer. *Cancer Res.*, **49**, 2087.

ZHOU, D., BATTIFORA, H., YOKATA, J., YAMAMOTO, T. & CLINE, M.J. (1987). Association of multiple copies of the c-*erb*B-2 oncogene with spread of breast cancer. *Cancer Res.*, **47**, 6123.